# A tool for investigating the differential functions of aggressive behavior in the face‐to‐face and cyber context: Extending the Cyber‐Aggression Typology Questionnaire

**DOI:** 10.1002/ab.21894

**Published:** 2020-05-07

**Authors:** Daniel Graf, Takuya Yanagida, Albert Maschler, Christiane Spiel

**Affiliations:** ^1^ Department of Developmental and Educational Psychology, Faculty of Psychology University of Vienna Vienna Austria

**Keywords:** aggression, cyber‐aggression, face‐to‐face aggression, motivational valence, self‐control

## Abstract

Aggressive behavior in the face‐to‐face and cyber contexts is driven by underlying aggression (i.e., functions of aggressive behavior). Common theories of aggression distinguish between reactive (e.g., rage) and proactive (e.g., seeking to achieve power and affiliation) aggression. However, according to the quadripartite violence typology, this distinction conflates aspects of motivational valence with self‐regulatory processes. The Cyber‐Aggression Typology Questionnaire (CATQ; Runions et al., 2017, *Aggress Behav*, 43(1), pp. 74–84) overcomes this weakness by identifying four types of cyber‐aggression (impulsive‐aversive/rage, controlled‐aversive/revenge, controlled‐appetitive/reward, and impulsive‐appetitive/recreation cyber‐aggression). However, the CATQ only considers aggression in cyberspace. We extended the CATQ to the face‐to‐face context by developing a corresponding Face‐to‐Face Aggression Typology Questionnaire (FATQ). The aim of this study was to investigate factorial and convergent validity and metric measurement invariance between four‐factorial cyber and face‐to‐face aggression. In total, 587 students from six Austrian universities filled out the CATQ, the FATQ, and additional scales during regular university lectures to examine convergent validity. Confirmatory factor analysis supported the four‐factor structure of both questionnaires, after excluding inconclusive items from the impulsive‐aversive/rage subscale of the FATQ. These items were also removed from the CATQ to obtain two symmetric questionnaires. Metric measurement invariance between the CATQ and the FATQ was confirmed. Convergent validity was largely observed. Our results support an extended four‐factor model of aggression. Having two parallel questionnaires, the FATQ and CATQ, enables future studies to investigate commonalities and differences in underlying drivers of aggressive behavior in the cyber and face‐to‐face contexts.

## 1 INTRODUCTION

Investigating humans’ motivations for engaging in aggressive behavior provides important knowledge for developing evidence‐based prevention and intervention strategies. However, reasons to engage in aggressive behavior are manifold (Runions, Bak, & Shaw, 2017). Moreover, according to the general aggression model, which assumes that “situational factors influence aggression by influencing cognition, affect, and arousal” (Anderson & Bushman, 2002, p. 37), aggressive behavior may be exhibited for different reasons in different contexts (e.g., face‐to‐face, cyberspace). This assumption is underpinned by studies finding perceived differences in context‐inherent properties of the face‐to‐face and cyber contexts (e.g., differences in perceived anonymity, lack of authorities, immediate reactions by others, audience size; see Graf, Yanagida, & Spiel, 2019). With respect to bullying as a subset of aggression (Smith, 2004), studies have repeatedly shown that motives for bullying via information and communication technologies (ICTs), more commonly known as cyberbullying, are partially different than motives for face‐to‐face bullying. Frequently discussed motives for cyberbullying are recreation, fun‐seeking, and revenge (Compton, Campbell, & Mergler, 2014; Gradinger, Strohmeier, & Spiel, 2012; Raskauskas & Stoltz, 2007), while face‐to‐face bullying is more commonly associated with rage, frustration, power and affiliation motives (Dodge & Coie, 1987; Roland & Idsøe, 2001; Schwartz et al., 1998). Hence, research on aggression must deal with the heterogeneity of the functions of aggressive behavior as well as the different contexts (face‐to‐face, cyberspace) in which aggressive behavior appears. However, to the best of our knowledge, no instrument exists at this juncture that assesses both aspects simultaneously.

### 1.1 The functions of aggressive behavior

The functions of aggressive behavior have mostly been discussed in the literature distinguishing between reactive and proactive aggression (e.g., Dodge, 1991; Dodge & Coie, 1987; Schwartz et al., 1998). The concept of reactive aggression is based on frustration‐anger theory (e.g., Berkowitz, 1993) and often referred to as “hot‐blooded,” or anger‐related, impulsive, retaliatory and provoked aggression. The concept of proactive aggression is derived from social learning theory (e.g., Bandura, 1983) and is typically seen as unprovoked and reward‐related aggression in connection with social rewards such as social status and positive affiliations with other aggressors (e.g., Fandrem, Strohmeier, & Roland, 2009; Roland & Idsøe, 2001; Schwartz et al., 1998). Self‐report measures based on this dichotomous distinction, such as the Reactive‐Proactive Aggression Questionnaire (RPQ; Raine et al., 2006) and the Impulsive/Premediated Aggression Scales (Stanford et al., 2003), have been used extensively in aggression and bullying research.

More recently, Howard (2011) criticized the dichotomous reactive‐proactive distinction for conflating motivational valence (aversive vs. appetitive) and self‐control (impulsive vs. controlled). For instance, the RPQ partially operationalizes proactive aggression by asking how often the respondent has “hurt others to win a game” (Raine et al., 2006, p. 170). While this item appropriately captures motivational valence (i.e., appetitive, reward, and status‐related), it does not explicitly consider whether the aggressive act was elicited spontaneously or planned, but implicitly assumes that proactive aggression is always conducted in a controlled manner. Similarly, with respect to reactive aggression, the RPQ asks, for example, how often the respondent has “hit others to defend himself” (Raine et al., 2006, p. 170). Here again, the motivational valence (i.e., aversive) is explicitly considered, but the level of self‐control (i.e., does the reaction follow immediately or with a time delay) remains unclear. Instead, a lack of self‐control is implicitly assumed (for details, see Howard, 2011).

To address these demonstrated shortcomings, Howard (2011) proposed the quadripartite violence typology (QVT). The QVT disentangles the functions of aggressive behavior by considering motivational valence (aversive vs. appetitive) and self‐control (impulsive vs. controlled) as two orthogonal dimensions (Howard, 2011). Hence, according to Howard (2011), aggressive behavior can have four distinct underlying functions: impulsive‐aversive aggression, controlled‐aversive aggression, controlled‐appetitive aggression, and impulsive‐appetitive aggression. Simplifying this nomenclature, Runions et al. (2017) proposed an alternative wording that refers more concretely to the motives of aggressive behavior, terming impulsive‐aversive aggression “rage aggression”, controlled‐aversive aggression “revenge aggression”, controlled‐appetitive aggression “reward aggression”, and impulsive‐appetitive aggression “recreation aggression”. Hereinafter, we will use the nomenclature proposed by Runions et al. (2017). According to Howard (2011) and Runions et al. (2017), the motive for rage aggression is to immediately eradicate a threat or frustration; the motive for revenge aggression is to take vengeance; the motive for reward aggression is the desire for positive outcomes such as power and affiliation (see e.g., Roland & Idsøe, 2001); and the motive for recreation aggression is thrill‐seeking and fun. Whereas rage aggression is closely related to reactive aggression and reward aggression to proactive aggression (Runions, Salmivalli, Shaw, Burns, & Cross, 2018), the QVT identifies recreation aggression and revenge aggression as additional distinct functions of aggressive behavior (see Figure S1).

### 1.2 Measuring the functions of aggressive behavior based on the QVT: Considering the context (face‐to‐face, cyberspace)

Based on the QVT, Bjørnebekk and Howard (2012) initially developed and validated the Angry Aggression Scales (AAS; 20 items) with samples of adolescents with and without conduct problems. Support was found for the postulated four‐factor structure as well as convergent and discriminant validity. Adolescents with conduct problems consistently exhibited higher scores for all four factors of aggression compared to adolescents without conduct problems. However, the context in which the aggressive behavior occurs was not considered (see Bjørnebekk & Howard, 2012). In light of this limitation, Runions et al. (2017) developed the Cyber‐Aggression Typology Questionnaire (CATQ) specifically for the cyber context by adapting items from the AAS to the cyber context and generating new ones. They validated the 29‐item CATQ with a sample of 314 undergraduate students, finding support for the postulated four‐factor structure and partial support for the scales’ convergent validity (see Runions et al., 2017).

In their work, Runions et al. (2017) emphasized the importance of context‐specific measurement tools. They reviewed studies investigating the relationship between aggressive behavior in cyberspace and the original versus cyber‐specific version (Law, Shapka, Domene, & Gagné, 2012) of the RPQ. Whilst applying the original version of the RPQ revealed that only proactive aggression was positively related to aggressive behavior in cyberspace (controlling for reactive aggression) (see Ang, Huan, & Florell, 2014; Calvete, Orue, Estévez, Villardón, & Padilla, 2010), a study using the cyber‐specific version of the RPQ found, on the contrary, a stronger association between reactive aggression and aggressive behavior in cyberspace (see Shapka & Law, 2013). Hence, it may be necessary to specify the context (face‐to‐face, cyberspace) in which aggressive behavior occurs when measuring aggression. However, to the best of our knowledge, no instrument enabling the simultaneous investigation of four‐factorial cyber and face‐to‐face aggression currently exists.

### 1.3 The present study

The general aim of the present study was to provide an instrument for measuring differential functions of aggressive behavior while considering face‐to‐face and cyber contexts. It consisted of four sub‐goals. With respect to construct validity, our first goal was to replicate the four‐factor structure of the CATQ with Austrian data.

Second, we adapted the CATQ items to the face‐to‐face context by carefully replacing the cues referring to cyberspace with cues that characterize the face‐to‐face context. We named the resulting questionnaire the Face‐to‐Face Aggression Typology Questionnaire (FATQ). As with the CATQ, we aimed to confirm the four‐factor structure of the FATQ.

Third, we tested the metric measurement invariance of the CATQ and the FATQ, which enables a comparison of factor variance and factor covariance across both instruments. That is, we assumed that corresponding items with matched item content referring to the cyber and the face‐to‐face context would have the same factor loadings.

Fourth and finally, we tested the convergent validity of the CATQ and the FATQ by investigating the associations between both instruments and the dichotomous measure of aggression (RPQ; Raine et al., 2006) as well as self‐rated manifestations along the behavioral inhibition and activation systems (BIS/BAS; according to Gray's psychobiological model of personality, 1987). We selected these two measures based on the original study (Runions et al., 2017). The BIS/BAS theory differentiates between two neural systems, with the BIS, which refers to sensitivity to punishment and fear and the BAS, which refers to reward sensitivity (Gray, 1987). It is suggested, that higher sensitivity to punishment and fear (BIS) results in higher vigilance, physiological activation and behavioral inhibition, whereas higher sensitivity to reward (BAS) reinforces ongoing behavior in the pursuit of a desired goal (Gray, 1987). In contrast to Runions et al. (2017) who assessed the BIS/BAS by applying the BIS/BAS scales (Carver & White, 1994), including subscales for BIS (assessed by one dimension) and BAS (Fun Seeking), BAS (drive), and BAS (Reward Responsiveness), we operationalized the BIS/BAS using the Action Regulating Emotion System Scale (ARES‐K; Hartig & Moosbrugger, 2003), with two subscales each for the BIS (anxiety and frustration) and the BAS (drive and gratification; see Hartig & Moosbrugger, 2003). We decided to use this conceptualization of Gray's model over the BIS/BAS scales (Carver & White, 1994), due to evidence of poor psychometric properties for the German version of the BIS/BAS scales (see Strobel, Beauducel, Debener, & Brocke, 2001). In this conceptualization, anxiety refers to negative affective reactions to anticipated aversive stimuli (equal to BIS in the BIS/BAS scales). The ARES‐K additionally considers frustration as a subdimension of the BIS, based on Gray's theorizing that the BIS is also involved in negative affective responses to the loss of reinforcement and thus nonachievement (Hartig & Moosbrugger, 2003). With respect to the BAS, drive is operationalized as momentum in the pursuit of a goal (equal to drive in the BIS/BAS scales) and gratification as joy in one's success (equal to reward responsiveness in the BIS/BAS scales). In contrast to the BIS/BAS scales (Carver & White, 1994), the ARES‐K does not include the need for new and stimulating situations (fun‐seeking in the BIS/BAS scales) as the scale's authors argue that this is conceptually more closely related to the concept of sensation seeking (Hartig & Moosbrugger, 2003).

With respect to the associations between four‐factor cyber and face‐to‐face aggression and the RPQ, we hypothesized positive associations between cyber and face‐to‐face rage aggression and the RPQ's reactive aggression measure. This is because both reactive aggression and rage aggression are operationalized as an uncontrolled aversive aggression. With respect to cyber and face‐to‐face revenge aggression, we expected positive relationships with both the reactive and proactive aggression measures of the RPQ. This is because revenge aggression both face‐to‐face and in cyberspace is assumed to be elicited by aversive stimuli (like reactive aggression) but conducted in a controlled manner (like proactive aggression). We expected cyber and face‐to‐face reward aggression to be positively associated with proactive aggression. This is due to the deliberate and reward‐related nature of both conceptualizations. Finally, we hypothesized positive associations between cyber and face‐to‐face recreation aggression and both reactive and proactive aggression. This was assumed because recreation aggression is reward‐related (like proactive aggression) yet impulsive (like reactive aggression).

We expected the following associations between the four factors of cyber and face‐to‐faceaggression and the behavioral inhibition and activation systems (BIS/BAS; Gray, 1987). We hypothesized negative relationships between cyber and face‐to‐face rage aggression and anxiety (BIS). This is due to the assumption that sensitivity to impending aversive stimuli (i.e., anxiety) might hinder the ability to act out impulsively (Hartig & Moosbrugger, 2003). Furthermore, we hypothesized associations between cyber and face‐to‐face rage aggression and frustration (BIS). However, we refrained from formulating a directed hypothesis here in light of uncertainty in the literature about the functional role of frustration in the behavioral inhibition system (Hartig & Moosbrugger, 2003). On the one hand, it is argued that sensitivity to frustration might play a behavioral inhibiting role, but on the other hand, it may also foster rage‐driven aggressive behavior (due to nonachievement of a goal). We expected positive associations between cyber and face‐to‐face revenge aggression and drive (BAS). This assumption is based on their common purposeful nature. Moreover, we hypothesized positive associations between cyber and face‐to‐face reward aggression and drive (BAS) as well as gratification (BAS). This is due to the common purposeful nature of cyber and face‐to‐face reward aggression and drive (BAS) and the common reward‐related nature of cyber and face‐to‐face reward aggression and gratification (BAS). Finally, we expected positive associations between cyber and face‐to‐face recreation aggression and gratification (BAS), in light of their shared reward‐related nature. See Figure S2 for a graphical representation of the hypotheses.

## 2 METHOD

### 2.1 Sample and procedure

A total of 587 university students (63.7% female; *M*_age_ = 21.85 years; *SD* = 4.18) from six Austrian universities answered online questionnaires via their smartphones during regular class time. A trained research assistant was present at all times. Participants gave informed consent and participation was voluntary. To avoid any systematic order effects, the order of the CATQ and the FATQ were randomly counterbalanced across participants.

To ensure linguistic equivalence, we translated and back‐translated the CATQ following common standards (see Beck, Bernal, & Froman, 2003; Hambleton, 2001; Peña, 2007). In the first stage, a trained research assistant with excellent English language competencies translated the English version of the CATQ into German. This first version was then back‐translated into English by an independent translation scientist. Afterward, a committee of academic psychologists in the area of aggression research assessed the semantic equivalence of the German and the English versions and made further adaptations. Finally, the original and back‐translated versions were compared with respect to wording (i.e., literal assessment) and meaning (i.e., semantic equivalence). Subsequently, the final items were adapted to the face‐to‐face context by all expert committee members separately by carefully replacing cues referring to the cyber context with cues referring to the face‐to‐face context. Meticulous attention was paid to retaining the residual semantic content of the items. Items were only accepted if all committee members agreed with their content. To give an example, we adapted the original item “If someone tries to hurt me, I will use my smartphone or the internet to get back at them in my own time” to “If someone tries to hurt me, I will get back at them face‐to‐face in my own time”.

### 2.2 Measures

Below, we present the measures used in this study.

#### 2.2.1 Four factors of cyber and face‐to‐face aggression (CATQ and FATQ)

In accordance with Howard's (2011) QVT, the 29‐item CATQ measures four distinct types of cyber‐aggression rooted in the dimensions of motivational valence and self‐control (Runions et al., 2017). Concretely, it assesses cyber rage aggression (12 items; sample item: “If someone tries to hurt me, I will use an ICT device to immediately get back at them”), cyber revenge aggression (6 items; sample item: “If someone tries to hurt me, I will use my ICT device to get back at them in my own time”), cyber reward aggression (6 items; sample item: “If I don't like someone, I use the internet to turn others against them”) and cyber recreation aggression (5 items; sample item: “If I'm having fun and joking online, I don't care if someone's feelings get hurt”).

Moreover, as previously discussed, we adapted all CATQ items to the face‐to‐face context, resulting in the FATQ. Thus, we considered two context‐specific questionnaires assessing four‐factorial cyber‐aggression as well as four‐factorial face‐to‐faceaggression. The items for the cyber context were presented after the following introduction: “The questions below are related to your behavior in cyberspace (e.g., communication on the internet: (group)chats, social media, forums, blogs, etc.)”, while the items for the face‐to‐face context were presented after the following introduction: “The following questions are exclusively related to your behavior in direct, personal contact (“face‐to‐face”)”. Answers to all items were given on a four‐point response scale (1 = *not at all true of me*, 2 = *partly true of me*, 3 = *fairly true of me*, 4 = *very true of me*. Ordinal Cronbach's *α* coefficients were .93/.87 (cyberspace/face‐to‐face) for rage aggression, .92/.84 (cyberspace/face‐to‐face) for revenge aggression, .94/.85 (cyberspace/face‐to‐face) for reward aggression, and .92/.88 (cyberspace/face‐to‐face) for recreation aggression.

#### 2.2.2 Reactive and proactive aggression (RPQ)

We measured the dichotomous conceptualization of reactive and proactive aggression with the RPQ (Raine et al., 2006). The RPQ consists of 23 items which measure reactive aggression (11 items; sample item: “How often have you hit others to defend yourself?”) and proactive aggression (12 items; sample item: “How often have you hurt others to win a game?”). Participants rated each item on a 3‐point response scale (1 = never, 2 = sometimes; 3 = often). Ordinal Cronbach's *α* coefficients were .86 for reactive aggression and .95 for proactive aggression. Confirmatory factor analysis (CFA) showed an acceptable model fit for the measurement model for the RPQ removing one item from the reactive aggression scale (*χ* ^2^(200) = 388.74, Comparative Fit Index [CFI] = .943, Tucker‐Lewis Index [TLI] = 0.934, root mean square error of approximation [RMSEA] = 0.040 and standardized root mean square residual [SRMR] = 0.079).

#### 2.2.3 BIS and BAS sensitivity (ARES‐K)

To assess the personality dimensions of BIS and BAS sensitivity, we used the 20‐item short version of the ARES‐K (Hartig & Moosbrugger, 2003). According to Hartig and Moosbrugger (2003), the BIS items on this scale refer to contexts characterized by cues indicating potential punishments or the absence of rewards as the original BIS/BAS scales (i.e., anxiety; Carver & White, 1994). Hartig and Moosbrugger (2003) additionally conceptualized a second dimension of the BIS that reflects sensitivity to a lack of reward (i.e., frustration). Thus, the ARES‐K operationalizes the BIS with two subscales: anxiety (5 items; sample item: “I quickly get nervous when I realize that I did something wrong”) and frustration (5 items; sample item: “If something does not go as well as I had hoped, I quickly get frustrated”). With respect to the BAS, two dimensions were assessed: drive and gratification. Drive refers to the intensification of ongoing actions in the face of achievable rewards, nonpunishments or goals (5 items; sample item: “If I have a goal in mind, it's hard to hold me down”). Gratification assesses joy upon achieving a goal (5 items; e.g., “It makes me very happy when I reach a desired goal”). Participants were asked to agree or disagree with each statement on a 4‐point response scale (1 = no, 2 = somewhat no, 3 = somewhat yes, 4 = yes). Ordinal Cronbach's α coefficients were .91 for anxiety, .91 for frustration, .81 for drive, and .84 for gratification. CFA showed an acceptable model fit for the measurement model for the ARES‐K (*χ* ^2^(165) = 737.22, CFI = .954, TLI = .947, RMSEA = .077 and SRMR = .079).

### 2.3 Missing data

A total of 0.17% of data were missing, stemming from 47 incomplete records. The percentage of missing values across the 101 variables ranged from 0.00% to 1.54%.

Full information maximum likelihood under the missing at random assumption was used to deal with the missing data (see Enders, 2010).

### 2.4 Analytic strategy

A series of CFAs (see Brown, 2015) based on factor models with ordered‐categorical indicators (see Bovaird & Koziol, 2012) using the robust weighted least squares estimator (WLSMV) were conducted in Mplus version 8.1 (Muthén & Muthén, 1998–2018) to investigate the present study's hypotheses. The measurement models were evaluated using the fit indices CFI, TLI, RMSEA, and SRMR based on common cut‐off criteria (see Kline, 2016).

First, measurement models for the four factors representing the four distinct types of cyber and face‐to‐face aggression were tested, before establishing a measurement model for the CATQ and the FATQ.

Next, metric measurement invariance between the CATQ and the FATQ was tested. To evaluate the equality of factor loadings, a measurement model with freely estimated factor loadings for the CATQ and the FATQ factors (i.e., configural invariance model) and a measurement model with factor loadings constrained to be equal between the CATQ and the FATQ factors (i.e., metric invariance model) was estimated. To compare the configural and the metric invariance model, changes in CFI and RMSEA were examined to evaluate the equality of factor loadings. It has been suggested that a change in CFI of more than .01 and a change in RMSEA of more than .015 (Cheung & Rensvold, 2002) indicate a meaningful decrease in model fit, making the invariance assumption about equal factor loadings not reasonable.

Last, the convergent validity of the CATQ and the FATQ was assessed by investigating the product‐moment correlation coefficients including 95% bias‐corrected bootstrap confidence interval based on 5,000 bootstrap draws (MacKinnon, 2008) between the CATQ, the FATQ and the RPQ and the ARES‐K scales.

## 3 RESULTS

### 3.1 Descriptive statistics

Response category proportions for all items of the CATQ and the FATQ scales are provided in Table S1. The item response distribution for the CATQ was positively skewed, with the largest proportion of students reporting disagreement with the content of each item. The item response distribution for the FATQ was less skewed than for the CATQ, especially for rage and revenge aggression.

### 3.2 Construct validity

To assess construct validity for both the CATQ and the FATQ, CFA was conducted.

#### 3.2.1 Construct validity: CATQ

Results of the CFA showed a very good model fit for the rage, revenge, reward, and recreation aggression scales (see Table S2). Accordingly, the four‐factor measurement model exhibited a very good model fit (*χ* ^2^(367) = 886.68, CFI = .952, TLI = 0.947, RMSEA = 0.049, and SRMR = 0.068). Measurement items and standardized factor loadings are shown in Table S3.

#### 3.2.2 Construct validity: FATQ

Results of the CFA showed a very good model fit for the revenge, reward, and recreation aggression scales (see Table S2). However, the measurement model for the rage aggression scale did not exhibit an acceptable model fit (*χ* ^2^(45) = 414.73, CFI = .936, RMSEA = .118, and SRMR = .077). Subsequently, standardized factor loadings and modification indices were inspected to identify items with poor psychometric properties. Inspecting the item contents revealed that both Item 6 (“I overreact before I have a chance to think about the consequences when someone says something mean face‐to‐face”) and Item 10 (“If somebody criticizes me face‐to‐face, I often react aggressively without thinking of the consequences”) contained information about additional cognitive processes (i.e., thinking about consequences). Similarly, Item 7 (“If I get to know something in personal contact that gets me angry, I react too quickly and then regret the way I responded”) and Item 11 (“I hastily respond to something said in personal contact and regret it later”) also contained information about additional cognitive processes (i.e., regret). However, inspecting the content of Item 4 (“If someone makes me angry face‐to‐face, I quickly spread mean rumours in personal contact”) indicated that spreading mean rumours might be not an appropriate example of impulsively‐driven behavior in the face‐to‐face context, despite being common in cyberspace. As a result, Items 4, 6, 7, 10, and 11 were removed from the measurement model. We discuss the incompatibility of these items when transferred from the cyber to the face‐to‐face context more detailed in Section 4. After deleting these items, the modified measurement model for rage aggression showed a very good model fit (see Table S2). Accordingly, the four‐factor measurement model exhibited good model fit (*χ* ^2^(242) = 712.97, CFI = .944, TLI = 0.936, RMSEA = .058, and SRMR = .078). Measurement items and standardized factor loadings are provided in Table S4.

To ensure comparability between the CATQ and the FATQ, Items 4, 6, 7, 10, and 11 were also removed from the cyberspace version of the questionnaire resulting in a modified version of the CATQ (mCATQ). The model fit for the reduced four‐factor measurement model exhibited a very good model fit (*χ* ^2^(242) = 506.38, CFI = .971, TLI = .967, RMSEA = .043 and SRMR = .058).

### 3.3 Measurement invariance

The results of the CFA for the measurement model comprising four factors of the FATQ and four factors of the mCATQ revealed a good model fit for the configural invariance model (*χ* ^2^(252) = 1800.15, CFI = .948, TLI = .943, RMSEA = .036 and SRMR = 0.074) and the metric invariance model (*χ* ^2^(232) = 1856.57, CFI = .946, TLI = .941, RMSEA = .037 and SRMR = .080). Moreover, there was no meaningful decrease in model fit between the configural and metric invariance models (see Table S5). Thus, it can be assumed that factor loadings are invariant across the mCATQ and the FATQ.

### 3.4 Convergent validity

To assess convergent validity, correlations including 95% bias‐corrected bootstrap confidence interval between both the mCATQ and the FATQ and the RPQ and the ARES‐K were investigated. All predicted relationships between the RPQ and the mCATQ and the FATQ were found (see Table 1). The expected results regarding the associations between the ARES‐K and the mCATQ and the FATQ could largely not be confirmed (see Table 1). More specifically, contrary to our assumptions, neither cyber nor face‐to‐face rage aggression was negatively related to anxiety, 95% CI [−.01, .14] and [−.16, .00] respectively. Cyber rage aggression was positively associated with frustration as expected [.07, .23], but face‐to‐face rage aggression was not [−.01, .16]. Moreover, we could not confirm the hypothesized positive relationships between cyber and face‐to‐face revenge aggression and drive, 95% CI [−.21, .03] and [−.00, .16] respectively; between cyber and face‐to‐face reward aggression and drive, 95% CI [−.30, −.02] and [−.19, −.02] respectively; between cyber and face‐to‐face reward aggression and gratification, 95% CI [−.33, −.10] and [−.24, −.07] respectively; or between cyber and face‐to‐face recreation aggression and gratification, 95% CI [−.27, −.04] and [−.14, .03] respectively. We discuss these findings in the next section.

**Table 1:** Correlations including 95% bias‐corrected bootstrap confidence interval based on 5,000 bootstrap draws between CATQ, FATQ, RPQ, and ARES‐K

Scale	1.	2.	3.	4.	5.	6.	7.	8.	9.	10.	11.	12.	13.	14.
1. CATQ Rage														
2. CATQ Revenge	.74													
	[.68, .79]													
3. CATQ Reward	.50	.60												
	[.39, .59]	[.47, .69]												
4. CATQ Recreation	.44	.45	.72											
	[.32, .54]	[.30, .57]	[.60, .81]											
5. FATQ Rage	.34	.18	.05	.12										
	[.27, .41]	[.10, .25]	−[.01, .13]	[.05, .19]										
6. FATQ Revenge	.31	.36	.18	.14	.46									
	[.23, .39]	[.28, .43]	[.11, .26]	[.08, .21]	[.39, .52]									
7. FATQ Reward	.32	.38	.53	.45	.23	.41								
	[.22, .42]	[.27, .48]	[.42, .63]	[.34, .56]	[.16, .30]	[.34, .47]								
8. FATQ Recreation	.26	.27	.48	.64	.15	.22	.60							
	[.15, .37]	[.14, .40]	[.35, .59]	[.54, .72]	[.08, .22]	[.14, .29]	[.51, .68]							
9. RPQ Reactive Aggression	**.32**	**.31**	.26	**.25**	**.26**	**.29**	.41	**.32**						
	[.24, .39]	[.23, .39]	[.18, .36]	[.17, .34]	[.18, .34]	[.21, .36]	[.34, .47]	[.24, .39]						
10. RPQ Proactive Aggression	.27	**.30**	**.49**	**.39**	.09	**.14**	**.41**	**.37**	.56					
	[.15, .40]	[.17, .44]	[.29, .66]	[.24, .54]	[−.01, .17]	[.07, .22]	[.30, .52]	[.25, .49]	[.47, .64]					
11. ARES‐K Anxiety	.06^a^	.10	.03	−.12	−.08^a^	.09	.05	−.12	.22	−.03				
	[−.01, .14]	[.02, .17]	[−.10, .05]	[−.20, −.04]	[−.16, .00]	[.00, .17]	[−.03, .13]	[−.19, −.03]	[.14, .29]	[−.12, .07]				
12. ARES‐K Frustration	**.15**	.16	.01	−.05	.08^a^	.17	.13	−.04	.36	.04	.78			
	[.07, .23]	[.09, .24]	[−.06, .08]	[−.12, .02]	[−.01, .16]	[.08, .25]	[.05, .20]	[−.11, .03]	[.29, .43]	[−.05, .13]	[.74, .82]			
13. ARES‐K Drive	−.09	−.08^a^	−.15^a^	−.17	.21	.08^a^	−.10^a^	−.13	−.04	−.13	−.04	.01		
	[−.19, .02]	[−.21, .03]	[−.30, −.02]	[−.30, −.05]	[.13, .28]	[−.00, .16]	[−.19, −.02]	[−.23, −.05]	[−.13, .04]	[−.24, −.02]	[−.13, .05]	[−.09, .09]		
14. ARES‐K Gratification	−.11	−.11	−.21^a^	−.15^a^	.14	.05	−.15^a^	−.05^a^	−.11	−.15	−.07	−.06	.57	
	[−.20, .00]	[−.23, −.01]	[−.33, −.10]	[−.27, −.04]	[.05, .22]	[−.04, .14]	[−.24, −.07]	[−.14, .03]	[−.19, −.04]	[−.26, −.04]	[−.15, .03]	[−.15, .03]	[.49, .64]	

*Note: N* = 586. Results in line with the hypotheses are in boldface.

Abbreviations: ARES‐K, Action Regulating Emotion System Questionnaire; RPQ, Reactive‐Proactive Questionnaire.

^a^ Results not in line with the hypotheses.

## 4 DISCUSSION

The present study focused on the underlying motives of aggressive behavior. Building upon the QVT, proposed by Howard (2011) and adapted to the cyber context by Runions (2013), this study extended the CATQ to the face‐to‐face context. CFAs revealed the same four‐factor structure for cyber‐aggression as in the original study (see Runions et al., 2017). For face‐to‐face aggression, we identified inconclusive items in the rage aggression subscale that were not transferable between contexts (face‐to‐face, cyberspace). Carefully checking these items revealed that they do not focus exclusively on the affective valence and the impulsive nature of the behavior (like all of the other items in this subscale), but also refer to more complex cognitive processes (i.e., thinking about consequences, regret). The differential functioning of these items across contexts might be explained by findings supporting the notion that cognitive processes such as thinking about consequences and regret are less relevant in cyberspace compared to the face‐to‐face context. This is often attributed to differences in context‐inherent properties. For example, the higher anonymity, the lack of absence of authorities and delayed reactions of others in cyberspace (e.g., Graf et al., 2019) might weaken or inhibit thinking about consequences and regrets (Sourander et al., 2010; Suler, 2004). Moreover, one inconclusive item focused on spreading rumours as an impulsive act, which is easily possible in cyberspace but quite difficult in the face‐to‐face context. Whereas spreading rumours in the face‐to‐face context may fulfil the functions of demonstrating power, achieving good social relationships or exacting revenge, and therefore be conducted in a controlled manner, in cyberspace, the same behavior might also result from impulsively‐driven rage. As expected, excluding the context‐sensitive items enhanced the model fit for face‐to‐face aggression, resulting in a valid four‐factor structure for the FATQ.

We subsequently also excluded the problematic items from the CATQ to obtain two context‐specific but equivalent questionnaires. This should enable further research to compare whether and to what degree the underlying aggressive functions of aggressive behavior (e.g., face‐to‐face bullying, cyberbullying) differs across contexts (face‐to‐face, cyberspace). To examine, whether such comparisons could be validly done, metric measurement invariance between the FATQ and the modified CATQ was checked. Our results support this potential application. It can be assumed that factor loadings are invariant.

Regarding convergent validity, we were able to confirm all hypothesized relationships between all four dimensions of cyber and face‐to‐face aggression and the two dimensions of the RPQ and thus to replicate Runions et al.'s (2017) findings. However, again like in Runions et al.'s (2017) study, investigating the relationships with the behavioral inhibition and activation systems, in our case operationalized via the ARES‐K, delivered less promising results. More concretely, we only found a meaningful positive relationship between cyber rage aggression and frustration. This suggests that the sensitivity to frustration might foster rage‐driven aggressive behavior. However, the same cannot be said of face‐to‐face rage aggression. Hence, based on our results, the relationship between rage aggression and frustration seems to be more relevant in cyberspace than in the face‐to‐face context. A possible explanation for this is Runions’ (2013) argument, that the constant availability of a social outlet in cyberspace might enhance anger and failures of self‐control, which might in turn consolidate the relationship between frustration and cyber (but not face‐to‐face) rage aggression.

All other results were not in line with our hypotheses. Similar to Runions et al. (2017), we found no evidence of a negative association between cyber rage aggression and anxiety. Surprisingly, the confidence interval mostly pointed in a positive direction. Although face‐to‐face rage aggression was not related to anxiety in our data either, the confidence interval had a predominantly negative range. Very tentatively interpreted, this might indicate that sensitivity to impending aversive stimuli could potentially hinder rage‐driven aggressive behavior in the face‐to‐face context but not in cyberspace. Theoretically, this seems plausible in light of the contextual differences pointed out by Runions (2013). For example, the perceived cue paucity in cyberspace might veil cues that cause anxiety, which might in turn weaken the behavioral inhibiting effect of anxiety on rage‐driven aggressive behavior. Moreover, the perceived lack of authorities and higher anonymity in cyberspace (see Graf et al., 2019) might additionally diminish the inhibiting effect of anxiety on rage aggression in cyberspace compared to face‐to‐face settings.

In line with Runions et al.'s (2017) results, cyber revenge aggression and drive were not associated with one another in our study. Nevertheless, while we hypothesized a positive relationship, the confidence interval predominantly encompassed negative values. There was also a lack of association between face‐to‐face revenge aggression and drive. However, the confidence interval here predominantly pointed in a positive direction. At the risk of overinterpreting this result, one could consider that revenge goes along with the desire to get even (Howard, 2011). This goal might be accomplished more easily in cyberspace (e.g., by posting mean comments), an environment where accountability seems reduced (Dooley, Pyżalski, & Cross, 2009), while in the face‐to‐face context, a stronger intensification of ongoing actions (i.e., drive; see Hartig & Moosbrugger, 2003) might be needed to engage in revenge‐driven aggressive behavior.

In contrast to the positive association between cyber reward aggression and drive reported by Runions et al. (2017), we observed negative relationships in our study. We suspect that this may be a consequence of the different operationalization of the BIS/BAS used. Whereas drive is operationalized in the BIS/BAS scales (Carver & White, 1994) used by Runions et al. (2017) with a strong emphasis on willingness and effort (e.g., “When I want something, I usually go all‐out to get it”), the ARES‐K (Hartig & Moosbrugger, 2003) operationalizes drive with greater focus on goal‐related flow (e.g., “If I have a prospect of success, that fills me with energy”). As the success anticipated as a result of reward‐related aggressive behavior (i.e., status, power and affiliation, see Roland & Idsøe, 2001) depends on complex social dynamics (e.g., Simon & Nail, 2013) and is accompanied by risk‐taking (Graf et al., 2019), it is conceivable that the willingness and effort aspect might be positively related to reward aggression. However, as the flow aspect is more related to gaining motivation as a result of concrete success, this operationalisation might be understood as more related to concrete tasks, such as those required in school (e.g., experiencing flow while solving a math problem). From this perspective, the negative association found in our study seems plausible, especially considering that high aggression levels might result in lower school engagement (Mehta, Cornell, Fan, & Gregory, 2013).

In line with Runions et al. (2017), we found a negative relationship between cyber reward aggression and gratification (termed reward responsiveness in the BIS/BAS scales) although a positive association was expected. That is, the joy in achieving a goal was negatively associated with cyber reward aggression in both studies. The same association was also found for face‐to‐face reward aggression in our study. One explanation for these findings could be that the joy in achieving a goal may be the wrong focus with respect to reward aggression. For example, people who instrumentalize aggressive behavior to achieve rewards (i.e., status, power and affiliations) might be problematic regarding their reward anticipation but might have no impairments with respect to goal attainment. Moreover, people who do not derive sufficient hedonic value from achieving specific goals may be motivated to pursue other goals and work harder to get that joy. This might fuel the use of transgressive means such as aggressive behavior.

Finally, we observed a negative association between cyber recreation aggression and gratification and a nonrelationship between face‐to‐face recreation aggression and gratification, although positive relations were proposed. As recreation aggression refers to impulsive thrill‐seeking, it could be considered an intrapersonal motive, which might in turn make it independent of external influences such as reactions by others. Instead, recreation‐related aggressive behavior might predominantly relate to immediate pleasant arousal, resulting from the aggressive behavior per se. In contrast to our previous assumptions, achieving joy upon achieving an external goal (i.e., gratification) might not be decisive for this connection.

In summary, we found limited results with respect to CATQ and FATQ's convergent validity vis‐a‐vis the ARES‐K. Although Runions et al. (2017) also found ambiguous results and some of our results were in line with their findings, other findings diverged between the studies. We attribute this to the different operationalisations of the BIS/BAS. From our perspective, the ARES‐K places a stronger emphasis on behavioral inhibition and activation processes during ongoing actions (example item for the anxiety scale: “When I feel that something I am doing is going to go wrong, I quickly become anxious and insecure”), whereas the BIS/BAS scale items also include anticipatory components (example item for the anxiety scale: “If I think something unpleasant is going to happen I usually get pretty “worked up””). This might explain contradictory results such as the negative association between cyber reward aggression and anxiety found by Runions et al. (2017), compared to the nonassociation found in our study. With respect to convergent validity vis‐a‐vis the RPQ, our results are in line with Runions et al.'s (2017) previous findings and strongly support the convergent validity of both the CATQ and the FATQ.

### 4.1 Limitations and future directions

Our study is based on a variant sample of students from several universities, studying different subjects. However, additional research with school students is needed. In our sample, the severity of four‐factorial aggression in both the face‐to‐face and cyber contexts was low. Future studies should investigate samples based on a more representative population. University students only represent a specific subsample of young people (usually aged from 18 to 25 years). The focus on younger students is expected to increase overall face‐to‐face and cyber‐aggression, as it would be more in line with the population at greatest risk for face‐to‐face and cyber‐aggression (Kowalski, Giumetti, Schroeder, & Lattanner, 2014). Observing the same four‐factor structure in this younger sample would provide additional support to our findings. Moreover, while results were in favour of convergent validity vis‐a‐vis the RPQ, the BIS/BAS might not be adequate for investigating CATQ and FATQ's convergent validity. For example, the BIS/BAS is conceptualised to investigate inhibiting and activating systems of ongoing behavior without specifying whether this behavior is focused on specific tasks (e.g., solving a concrete problem) or social goals (e.g., getting cool friends, see Bardach et al., 2019). Future studies might consider adapting the BIS/BAS to social contexts, to more adequately address continuous and complex social situations. Additional constructs such as impulsivity or self‐control could be used to further test for the scales’ convergent validity. To examine the consistency of the investigated four factors of aggression in the face‐to‐face and cyber contexts, future studies should also examine the test‐retest reliability of both the CATQ and the FATQ as well as the stability of results over time which has been shown to be relatively low for bullying behavior (Bergsmann, Finsterwald, Strohmeier, & Spiel, 2011). Moreover, future studies should focus on the CATQ and FATQ's predictive validity to investigate whether the two questionnaires can predict and are predicted by relevant variables longitudinally. Such a study is currently being planned.

Overall, our study provides an opportunity for future research to investigate distinct individual differences in the functions of aggressive behavior while considering the context in which aggressive behavior occurs. This is relevant for prevention and intervention research, as it could enable different subgroups of face‐to‐face and cyber‐aggressors (or bullies) with different motivational patterns within and between contexts to be identified and provided with tailor‐made interventions.

## CONFLICT OF INTERESTS

The authors declare that there are no conflict of interests.

## Supporting information

Supporting information

Supporting information

Supporting information

Supporting information

Supporting information

Supporting information

Supporting information
