# Supplementary material for: A tool for investigating the differential functions of aggressive behavior in the face‐to‐face and cyber context: Extending the Cyber‐Aggression Typology Questionnaire
Source: Aggress Behav. 2020 May 7;46(5):380–90. doi: 10.1002/ab.21894 (PMC7496625; doi:10.1002/ab.21894)
Supplement: Supplementary file 1 — Supporting information [file AB-46-380-s001.docx]

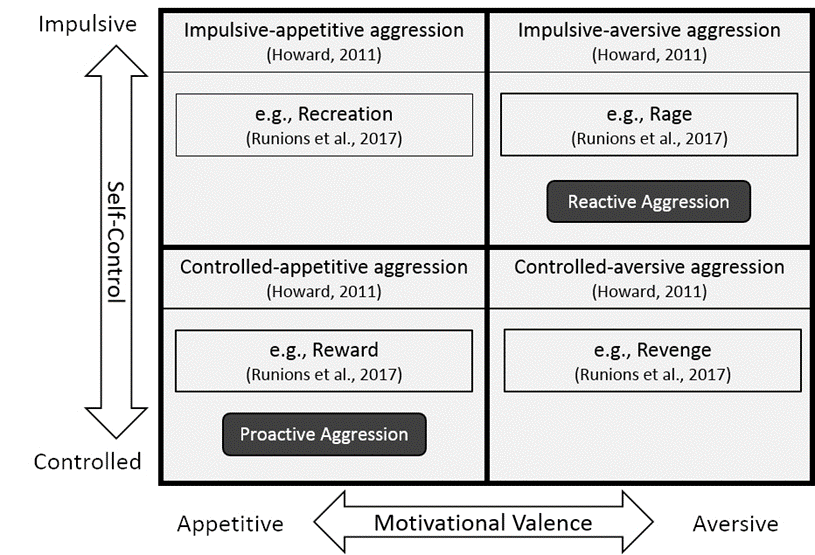


Figure 1. Quadripartite typology of aggression by Runions et al. (2017) based on Howard´s (2011) quadripartite violence typology
