# Supplementary figures and images for: A tool for investigating the differential functions of aggressive behavior in the face‐to‐face and cyber context: Extending the Cyber‐Aggression Typology Questionnaire
Source: Aggress Behav. 2020 May 7;46(5):380–90. doi: 10.1002/ab.21894 (PMC7496625; doi:10.1002/ab.21894)

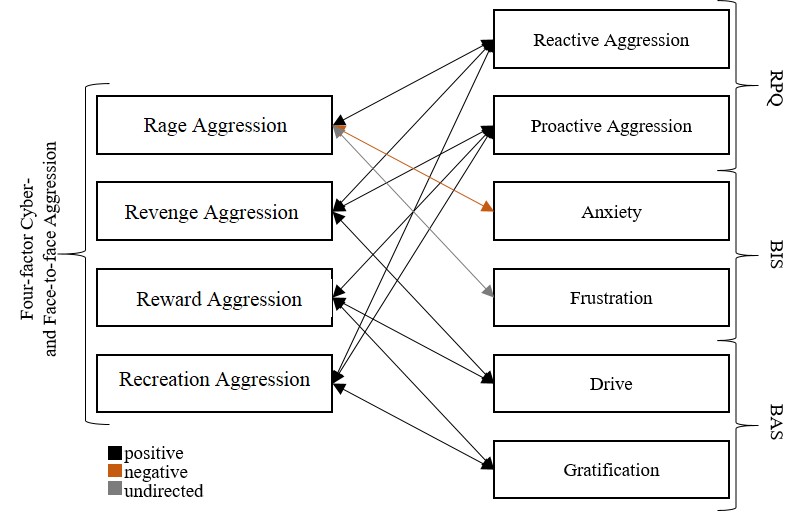


Figure 2. Hypotheses on convergent validity.

Supplement: Supplementary file 2 — Supporting information [file AB-46-380-s002.docx]
