# Supplementary material for: A tool for investigating the differential functions of aggressive behavior in the face‐to‐face and cyber context: Extending the Cyber‐Aggression Typology Questionnaire
Source: Aggress Behav. 2020 May 7;46(5):380–90. doi: 10.1002/ab.21894 (PMC7496625; doi:10.1002/ab.21894)
Supplement: Supplementary file 3 — Supporting information [file AB-46-380-s003.docx]

Table S1

*Response Category Proportions for CATQ and FATQ Items*

| Item | CATQ | | | |  | FATQ | | | |
| --- | --- | --- | --- | --- | --- | --- | --- | --- | --- |
|  | *Not at all true of me* | *Partly  true of me* | *Fairly true  of me* | *Very true  of me* |  | *Not at all true of me* | *Partly  true of me* | *Fairly true  of me* | *Very true  of me* |
| Rage | |  |  |  |  |  |  |  |  |
| Item 1  Item 2  Item 3  Item 4  Item 5  Item 6  Item 7  Item 8  Item 9  Item 10  Item 11  Item 12 | .800  .804  .689  .938  .799  .802  .754  .643  .628  .715  .710  .532 | .155  .140  .224  .046  .160  .152  .196  .224  .258  .246  .254  .315 | .038  .038  .046  .065  .007  .036  .039  .099  .084  .029  .026  .109 | .007  .010  .022  .009  .012  .010  .010  .034  .031  .010  .010  .044 |  | .070  .191  .145  .797  .265  .472  .420  .184  .140  .557  .376  .181 | .440  .490  .411  .162  .471  .393  .428  .350  .423  .321  .458  .396 | .355  .244  .316  .031  .208  .103  .114  .307  .309  .104  .137  .278 | .135  .075  .128  .010  .056  .032  .038  .159  .128  .017  .029  .145 |
| Revenge | |  |  |  |  |  |  |  |  |
| Item 1  Item 2  Item 3  Item 4  Item 5  Item 6 | .805  .836  .805  .889  .821  .691 | .150  .133  .156  .089  .123  .248 | .036  .026  .032  .015  .044  .046 | .009  .005  .007  .007  .012  .015 |  | .268  .408  .509  .474  .376  .442 | .462  .377  .357  .346  .364  .370 | .212  .179  .106  .128  .212  .142 | .058  .036  .029  .051  .048  .046 |
| Reward | |  |  |  |  |  |  |  |  |
| Item 1  Item 2  Item 3  Item 4  Item 5  Item 6 | .945  .956  .884  .913  .971  .889 | .038  .034  .084  .067  .015  .080 | .012  .003  .022  .017  .009  .019 | .005  .007  .010  .003  .005  .012 |  | .700  .884  .608  .508  .961  .681 | .215  .082  .285  .352  .024  .251 | .068  .022  .089  .111  .007  .053 | .017  .012  .019  .029  .009  .015 |
| Recreation | |  |  |  |  |  |  |  |  |
| Item 1  Item 2  Item 3  Item 4  Item 5 | .891  .829  .781  .918  .860 | .077  .137  .173  .053  .102 | .021  .019  .032  .015  .020 | .012  .015  .014  .014  .017 |  | .751  .542  .708  .817  .671 | .188  .344  .230  .133  .251 | .049  .085  .048  .034  .055 | .012  .029  .014  .015  .024 |
