# Supplementary material for: A tool for investigating the differential functions of aggressive behavior in the face‐to‐face and cyber context: Extending the Cyber‐Aggression Typology Questionnaire
Source: Aggress Behav. 2020 May 7;46(5):380–90. doi: 10.1002/ab.21894 (PMC7496625; doi:10.1002/ab.21894)
Supplement: Supplementary file 4 — Supporting information [file AB-46-380-s004.docx]

Table S2

Confirmatory Factor Analysis Results: CATQ and FATQ

| (Sub-) Scale | $\chi^{2}$ | df | CFI | TLI | RMSEA | SRMR |
| --- | --- | --- | --- | --- | --- | --- |
| CATQ | 506.38 | 242 | .971 | 0.967 | 0.043 | 0.058 |
| Rage | 54.03 | 12 | .986 | 0.975 | 0.077 | 0.036 |
| Revenge | 5.37 | 7 | 1.000 | 1.001 | 0.000 | 0.010 |
| Reward | 9.25 | 9 | 1.000 | 1.000 | 0.007 | 0.024 |
| Recreation | 10.87 | 5 | .997 | 0.994 | 0.045 | 0.016 |
| FATQ | 712.97 | 242 | .944 | 0.936 | 0.058 | 0.078 |
| Rage | 49.90 | 12 | .992 | 0.985 | 0.066 | 0.022 |
| Revenge | 20.25 | 7 | .993 | 0.985 | 0.057 | 0.019 |
| Reward | 8.96 | 9 | 1.000 | 1.000 | 0.000 | 0.021 |
| Recreation | 12.65 | 5 | .996 | 0.992 | 0.051 | 0.018 |

*Note*. *N* = 586; Items 4, 6, 7, 10, and 11 of the rage scale were excluded from the analysis.
