# Supplementary material for: A tool for investigating the differential functions of aggressive behavior in the face‐to‐face and cyber context: Extending the Cyber‐Aggression Typology Questionnaire
Source: Aggress Behav. 2020 May 7;46(5):380–90. doi: 10.1002/ab.21894 (PMC7496625; doi:10.1002/ab.21894)
Supplement: Supplementary file 5 — Supporting information [file AB-46-380-s005.docx]

Table S3

*Measurement Items and Factor Loadings for the CATQ*

| Item | SFL |
| --- | --- |
| **Rage: α = .91, CR = .93, AVE = .65**   1. If someone tries to hurt me, I will use an ICT device to immediately get back at them 2. If I get teased or threatened, I get angry easily and strike back online right away 3. I use ICT to get back at someone as soon as they post a hurtful message about me 4. If someone makes me angry online, I quickly post mean texts and messages online 5. If someone makes fun of me on the internet, I get frustrated and respond angrily online right away 6. I overreact before I have a chance to think about the consequences when someone says something mean online 7. If I see a message online that gets me angry, I react too quickly and then regret the way I responded 8. If someone tries to cyberbully me, I quickly lash back with something online 9. If someone says something online to hurt me, I post something back right away to get back at them 10. If somebody criticizes me online or in a text, I often react aggressively without thinking of the consequences 11. I hastily respond to something written online and regret it later 12. I respond very quickly to a message or post that is disrespectful to me   **Revenge: α = .92, CR = .92, AVE = .67**   1. If someone does something to hurt me, I would get back at them in my own time by using my ICT device(s) 2. If someone tries to hurt me, I will use my ICT device(s) to get back at them in my own time 3. I get back at people who make fun of me on the internet because their posts hurt more the more I think about them 4. I like using my ICT device(s) to plan my revenge when I feel angry at someone 5. If I need to get revenge on someone, I would rather strike back using my ICT device(s) where I can plan out how to do it 6. If I see a mean message about me on my ICT, it bothers me more and more when I think about it, and I try to get even   **Reward: α = .94, CR = .94., AVE = .74**   1. If I don’t like someone, I use the internet to turn others against him/her 2. Sometimes I’ll team up with my friends to bring someone down online 3. Sometimes I can be mean to people online to get what I want 4. When I don’t like a person, I use the internet to make them feel like they do not belong in my group 5. I pretend to be someone else online to ruin somebody else’s friendships 6. I have at times used the internet to make someone look like bad   **Recreation: α = .92, CR = .93, AVE = .73**   1. I get carried away having fun online and others think I’m being a cyberbully or a troll 2. I make fun of people I don’t know on the internet without thinking about whether they will see it or not 3. If I’m having fun and joking online, I don’t care if someone’s feelings get hurt 4. I repeatedly annoy people online because I think it’s funny 5. Joking online is so much fun that I don’t worry about whether someone might be bothered by what I say | 0.84  0.85  0.79  -  0.85  -  -  0.80  0.81  -  -  0.69  0.82  0.83  0.74  0.91  0.87  0.71  0.89  0.85  0.73  0.81  0.99  0.87  0.87  0.71  0.83  0.96  0.89 |

*Note*. English items from the original version are shown; Items 4, 6, 7, 10, and 11 of the rage scale were excluded from the analysis; SFL = standardized factor loading; α = Ordinal Cronbach’s alpha; CR = composite reliability; AVE = average variance-extracted.
