# Supplementary material for: A tool for investigating the differential functions of aggressive behavior in the face‐to‐face and cyber context: Extending the Cyber‐Aggression Typology Questionnaire
Source: Aggress Behav. 2020 May 7;46(5):380–90. doi: 10.1002/ab.21894 (PMC7496625; doi:10.1002/ab.21894)
Supplement: Supplementary file 6 — Supporting information [file AB-46-380-s006.docx]

Table S4

*Measurement Items and Factor Loadings for the FATQ*

| Item | SFL |
| --- | --- |
| **Rage: α = .87, CR = .87, AVE = .49**   1. If someone tries to hurt me in personal contact, I immediately get back at them 2. If I´m teased or threatened in personal contact, I get angry easily and strike back right away 3. I get back at someone face-to-face as soon as they spread something hurtful about me 4. If someone makes me angry face-to-face, I quickly spread mean rumours in personal contact 5. If someone makes fun of me in personal contact, I get frustrated and respond angrily face-to-face right away 6. I overreact before I have a chance to think about the consequences when someone says something mean face-to-face 7. If I get to know something in personal contact that gets me angry, I react too quickly and then regret the way I responded 8. If someone tries to bully me face-to-face, I quickly lash back with something in personal contact 9. If someone says something in personal contact to hurt me, I respond something back right away to get back at them 10. If somebody criticizes me face-to-face, I often react aggressively without thinking of the consequences 11. I hastily respond to something said in personal contact and regret it later 12. I respond very quickly to actions that are disrespectful to me   **Revenge: α = .84, CR = .82, AVE = .44**   1. If someone does something to hurt me, I would get back at them in my own time in personal contact 2. If someone tries to hurt me, I will get back at them in my own time face-to-face 3. I get back at people who make fun of me in personal contact because their deeds hurt more the more I think about them 4. I plan my revenge before I get back in personal contact, when I feel angry at someone 5. If I need to get revenge on someone, I would rather strike back face-to-face where I can plan out how to do it 6. If I find out something mean about me in personal contact, it bothers me more and more when I think about it, and I try to get even   **Reward: α = .85, CR = .86, AVE = .51**   1. If I don’t like someone, I use personal contact with others to turn them against him/her 2. Sometimes I’ll team up with my friends to beat somebody up in personal contact 3. Sometimes I can be mean to people in personal contact to get what I want 4. When I don’t like a person, I try to make them feel like they do not belong in my group through my behavior in personal contact 5. I pretend to be someone else to ruin somebody else’s friendships 6. I have at times used personal contact to make someone look like bad   **Recreation: α = .88, CR = .89, AVE = .63**   1. I get carried away having fun in personal contact and others think I’m being a bully or a troll 2. I make fun of people I don’t know in company without thinking about whether they will notice it or not 3. If I’m having fun and joking in personal contact, I don’t care if someone’s feelings get hurt 4. I repeatedly annoy people in personal contact because I think it’s funny 5. Joking in personal contact is so much fun that I don’t worry about whether someone might be bothered by what I say | 0.62  0.68  0.72  -  0.59  -  -  0.85  0.81  -  -  0.61  0.62  0.72  0.70  0.60  0.69  0.63  0.75  0.74  0.66  0.56  0.86  0.70  0.71  0.65  0.86  0.83  0.88 |

*Note*. English translations of the German items are shown; Items 4, 6, 7, 10, and 11 of the rage scale were excluded from the analysis; SFL = standardized factor loading; α = Ordinal Cronbach’s alpha; CR = composite reliability; AVE = average variance-extracted.
