# Supplementary material for: A tool for investigating the differential functions of aggressive behavior in the face‐to‐face and cyber context: Extending the Cyber‐Aggression Typology Questionnaire
Source: Aggress Behav. 2020 May 7;46(5):380–90. doi: 10.1002/ab.21894 (PMC7496625; doi:10.1002/ab.21894)
Supplement: Supplementary file 7 — Supporting information [file AB-46-380-s007.docx]

Table S5

*Summary of Model Fit Indices for Testing Measurement Invariance between the CATQ and the FATQ*

| Model | $\chi^{2}$ | df | CFI | ΔCFI | RMSEA | ΔRMSEA | SRMR |
| --- | --- | --- | --- | --- | --- | --- | --- |
| Configural Invariance | 1800.15 | 252 | .948 |  | 0.036 |  | 0.074 |
| Metric Invariance | 1856.57 | 232 | .946 | -0.002 | 0.037 | 0.001 | 0.080 |

*Note.* *N* = 586; ∆CFI = CFI_nested_ – CFI_previous_ (negative values indicate a decrease in model fit); ∆RMSEA = RMSEA _nested_ – RMSEA _previous_ (positive values indicate a decrease in model fit).
